# Range expansion and habitat shift triggered elevated diversification of the rice genus (*Oryza*, Poaceae) during the Pleistocene

**DOI:** 10.1186/s12862-015-0459-1

**Published:** 2015-09-03

**Authors:** Li Lin, Liang Tang, Yun-Jun Bai, Zhi-Yao Tang, Wei Wang, Zhi-Duan Chen

**Affiliations:** State Key Laboratory of Systematic and Evolutionary Botany, Institute of Botany, Chinese Academy of Sciences, 20 Nanxincun, Xiangshan, Beijing, 100093 China; University of Chinese Academy of Sciences, 52 Sanheli Road, Beijing, 100049 China; College of Horticulture and Landscape Architecture, Southwest University, 2 Tianhe Road, Beipei Distinct, Chongqing, 400715 China; Department of Ecology, College of Urban and Environmental Sciences and Key Laboratory for Earth Surface Processes, Peking University, 5 Yiheyuan Road, Haidian Distinct, Beijing, 100871 China

## Abstract

**Background:**

The rice genus (*Oryza*) contains many wild genetic resources that are vital to the well-being of humans. However, little is known about the process by which the genus diversified or the factors that drove its speciation. Here, we integrated the phylogenetic, molecular dating and biogeographic methods to investigate the spatial-temporal patterns of *Oryza* diversification, and used a series of model tests to examine whether intercontinental migrations and/or key innovations were associated with significant changes in diversification rates in the genus.

**Results:**

*Oryza* became differentiated in tropical Asia in the Miocene. There were two migrations from the ancestral area into Africa and Australia during the Miocene. We inferred at least 10 migration events out of tropical Asia since the Pleistocene, mainly involving the species adapting open habitat. A rapid increase in diversification rates of the whole *Oryza* occurred during the Pleistocene. Intercontinental migrations from tropical Asia to other tropical regions were positively correlated with shift in habitat, but not with changes in life history. A habitat preference shift from shade tolerant to open habitat predated the burst in diversification rates.

**Conclusions:**

Rice species may have been pre-adapted to invade open habitat. Significant increase in diversification rates occurred during the Pleistocene and is associated with range expansion and habitat shift, but not with life history. The rice genus provides an excellent case supporting the idea that range expansion and invasion of novel habitats can drive the diversification of a group.

**Electronic supplementary material:**

The online version of this article (doi:10.1186/s12862-015-0459-1) contains supplementary material, which is available to authorized users.

## Background

Understanding speciation processes is fundamental in ecology and evolutionary biology [1, 2]. An accurate estimation of how biodiversity evolved through time can enable us to better predict changes in future biodiversity [3]. Furthermore, understanding the diversification patterns and potential driving factors of a group with important wild genetic resources is vital for the well-being of humans and has far-reaching implications for the work of policy-makers and conservation biologists.

The rice genus (*Oryza*) is of economic importance due to Asian cultivated rice (*Oryza sativa* L.), which provides food for more than half of the world’s population. It was domesticated from wild relatives [4] that provide important resources of vital germplasm for genetic improvement to increase the production and quality of rice. *Oryza* has become an important model system for genetic and genomic studies [5]. Due to human activity or other unknown causes, some wild species in *Oryza* have become endangered or their population sizes have declined [6].

*Oryza* consists of 26 species and is represented by 10 genome groups (i.e. the A-, B-, C-, BC-, CD-, E-, F-, G-, HJ-, and HK-genomes; [7–9]; Additional file 1: Table S1). The phylogenetic relationships of the rice species have been robustly inferred (e.g. [10–12]). However, the diversification patterns of the genus and the factors that drove it remain unclear. Divergence time estimates have suggested several rapid diversification events in *Oryza* across different genome lineages and temporal scales. Zou et al. [13] hypothesized that the rice genus experienced two early rapid speciation events at ca. 15 million years ago (Ma) and ca. 5–6 Ma, respectively. Zhu and Ge [14] hypothesized that the A-genome lineage radiated at ca. 2 Ma. It is necessary to test these hypothetical rapid diversification events using more rigorous model-based diversification analysis methods.

**Fig. 1:**
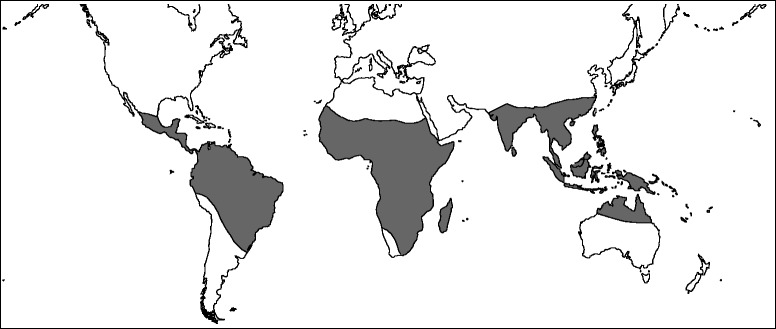
The Geographic distribution of *Oryza* wild species

The rice species are primarily restricted to tropical and subtropical regions, and the large majority of them are endemic to specific regions (Fig. 1; Additional file 1: Table S1). Recent studies indicate that *Oryza* originated in Asia [12, 15, 16], and that long-distance, transoceanic dispersals are responsible for its current distribution [12, 14, 15]. However, the extent to which dispersal events have contributed to the diversification of *Oryza* remains unclear. Meanwhile, rice species utilize shade and open habitats and demonstrate both annual and perennial life histories ([4, 9]; Additional file 1: Table S1). The origin of the annual *Oryza nivara* from a perennial ancestor, *Oryza rufipogon*, due to habitat differentiation, is a good example of a speciation event driven by the evolution of an ecological trait [17]. It remains unknown whether habitat and/or life history have been involved in the diversification process of *Oryza* or whether they are responsible for its current diversity. Newly developed model-based methods have been powerful tools for examining the diversification patterns of organisms and their underlying mechanisms (e.g. [18–21]); these model-based methods can reveal the processes that fostered the diversification of rice and shaped spatial-temporal patterns of its diversity.

Two Miocene macrofossils of Oryzeae have been reported [22, 23] and have been used in previous dating analyses of Oryzeae [12] or *Oryza* [13]. Their absolute ages are unknown, limiting their applicability. Recently, phytolith fossils from the late Cretaceous were assigned to the stem group of Oryzeae [24]. A subsequent study indicates that their use as a divergent calibration point strongly affects estimates of the divergence times of Poaceae [25]. However, their influence on the inferred ages of *Oryza* needs to be evaluated.

Here, we first reconstruct a comprehensive phylogeny for *Oryza* based on 20 plastid DNA regions, and then investigate the temporal and spatial patterns of rice diversification and its driving forces. We test two hypotheses: (1) range expansions have played an important role in the diversification of *Oryza*; and (2) habitat and/or life history shifts are associated with rice diversification.

## Methods

### Molecular sequence data

Previous studies have indicated that plastid and nuclear data recovered the similar phylogenetic relationships among different genome types and species in *Oryza* [10–12]. DNA sequences for twenty plastid regions were obtained from our previous studies [12] or GenBank (Additional file 1: Table S2). Our extensive taxon sampling encompasses 27 accessions, representing all currently recognized 16 diploid species and 10 tetraploid species of the genus. We also sampled 16 species representing the 11 other genera of Oryzeae. Following Tang et al. [12], we selected *Ehrharta erecta* (Ehrharteae) and *Phyllostachys aurea* (Bambusoideae) as outgroups. The sampled species and their GenBank accession numbers are listed in Additional file 1: Table S2.

### Phylogenetic analysis

Sequences were aligned in MUSCLE v3.7 [26] and manually adjusted in BioEdit v7.0.5.3 [27]. All DNA regions were aligned separately and concatenated before analyses. Phylogenetic analyses were conducted using Bayesian inference (BI) and maximum likelihood (ML) methods in MrBayes v3.1.2 [28] and RAxML v7.0.4 [29], respectively. Akaike Information Criterion (AIC) via Modeltest v3.06 [30] was used to determine the best-fit model for each partition (below). For BI analyses, we partitioned data *a priori* based on gene identity and general biochemical or evolutionary constraints. Four partitioning strategies were used: (1) combination of all data; (2) data partitioned into coding and noncoding regions; (3) one partition each for intron, spacer, and coding regions; and (4) one partition for each of the 20 regions. Bayes factors (BF) [31] were used to compare the different partitioning strategies and were calculated from post burn-in harmonic means of likelihood values from appropriate partitioned Bayesian phylogenetic analyses [32]. The fourth partitioning strategy was identified as optimal for our data and was applied in all subsequent analyses.

Four chains of the Markov chain Monte Carlo (MCMC) were run, sampling one tree every 1000 generations for 30 million generations, starting from a random tree, with the option *prset ratepr* set as variable. We used Tracer v1.5 [33] to evaluate whether the analysis converged. Majority rule (>50 %) consensus trees were constructed after removing the burn-in period samples (initial 25 % of the sampled trees). RAxML was run on the above optimal 20 partitions, each partition with a GTR + I + Γ model and all model parameters estimated, executing 1000 rapid bootstrap inferences before a thorough ML search.

### Divergence-time estimate

Divergence times were estimated in BEAST v1.7.4 [34], which employs a Bayesian MCMC approach to co-estimate topology, substitution rates and node ages. To evaluate the impact of phytolith fossils reported by Prasad et al. [24] to calibration of *Oryza*, we devised two calibration scenarios based on the different constraints for the stem age of Oryzeae. In scenario #1, we used three macrofossils as calibration points (Additional file 2: Figure S1). (i) The stem group node of Oryzeae was given a lower bound of 66 Ma, based on the phytolith fossils of Oryzeae from the Late Cretaceous (66–67 Ma) in India [24]. (ii) The crown age of *Leersia* was given a lower bound of 5.3 Ma, because the silicified anthoecia fossil from the Miocene found in North America shared some common features with the extant *Leersia ligularis* [23]. (iii) The crown age of *Oryza* was given a lower bound of 5.3 Ma, because the spikelet fossil from the Miocene found in Germany [22] shared some morphological features with the extant *Oryza granulata* (see [12]). Given the potential uncertainty of fossil calibrations in phylogenetic position, the time lapse between divergence from the extant lineage and fossilization, and geological dating, the use of nonuniform priors is sometimes problem when parameterization [35–37]. Here we used the uniform priors for these three fossil calibration points. In scenario #2, we replaced the 66-Ma stem group age of *Oryzeae* with 38.5 Ma based on the results of Christin et al. [25], which was set using a normal distribution (SD = 1.99).

All dating analyses were performed under the GTR + G + I model (with eight rate categories), a Yule tree prior, with rate variation across branches uncorrelated and lognormally distributed. MCMC chains were run for 30 million generations, sampling every 1000 generations. Tracer v1.5 [33] was used to assess appropriate burn-in and the adequate effective sample sizes of the posterior distribution (>200). The maximum clade credibility (MCC) tree with median branch lengths and a 95 % highest posterior density (HPD) interval on nodes was reconstructed using TreeAnnotator v1.5.4 [34].

### Biogeographical inference

To infer ancestral distributions on the phylogeny of *Oryza*, two methods were used: a parsimony-based method (S-DIVA; [38]) and a ML-based method (Lagrange v2.0.1; [39]). The S-DIVA analysis can take into account phylogenetic uncertainty [38]. We randomly sampled 1000 BEAST trees and used the MCC tree derived from the MCMC stationary sample as a final representative tree. The ML-based method uses the information contained in genetic branch lengths and allows changing dispersal probabilities across areas and time to be incorporated. Because the availability of movement corridors between continents is less well understood for *Oryza* [15], we did not employ geological constraints. We ran ML-based biogeographical inferences using two chronograms obtained in two calibration scenarios.

Distribution data for *Oryza* were compiled from the literature [4, 8, 9]. Five areas were scored by continental divisions and climate: tropical Asia (south of 25° N lat.), temperate Asia (north of 25° N lat.), Australia, Africa, and Americas (Fig. 1). Following Brummitt [40], New Guinea was assigned to tropical Asia. In *Oryza*, 23 of the 25 wild species are only restricted in one area, two in two areas (Additional file 1: Table S1). There are two cultivated species in *Oryza*, *O. glaberrima* (African cultivated rice) and *O. sativa* (Asian cultivated rice). The former is restricted to Africa, while the latter has been confirmed to be domesticated in tropical Asia [41]. Ancestral areas were inferred with the “maxareas” constrained to 2, reflecting the maximum number of areas for all 26 species.

### Diversification rate analysis

We run a series of diversification rate tests to identify the potential diversification rate shifts using two chronograms obtained in two calibration scenarios, respectively. Outgroups were pruned for the diversification rate analyses. The two genome groups (BB and BBCC) of *Oryza punctata* are distributed in different clades ([12]; this study), we here considered them as two individual species. Shifts in diversification rates of *Oryza* were investigated by inspecting lineage-through-time (LTT) plots. LTT plots were generated using R package APE v3.1 [42], for the 1000 randomly selected BEAST trees and for the MCC tree. We further used TreePar [20] to identify the locations of temporal shifts in diversification rates of *Oryza*. TreePar analyses were run with a grid setting of 0.1 million years with Yule and birth-death processes, respectively. Rate shifts were recognised as significant when *p* < 0.05 using the likelihood ratio test.

Bayesian analysis of macro-evolutionary mixtures (BAMM; [21]) was also used to infer speciation rates across the phylogeny of *Oryza*. The analyses were run on randomly sampled 100 BEAST trees. We ran BAMM for 10 million generations and discarded the first 20 % as burn-in after checking for convergence. We used the R package BAMMtools [43] to estimate rate-through-time dynamics and number of evolutionary regime shifts from the posterior sampling.

### Ancestral states and correlates of diversification

We investigated how two ecological characters, habitat and life history, were associated with increased rates of diversification in *Oryza*. Data were obtained from the World Grass Genera database [44] and the taxonomic literature [4, 7–9]. Based on light conditions of habitats of extant species, *Oryza* habitats can be divided into two types: close and open (Additional file 1: Table S1). Open habitat species grow in environments without a canopy of other plants, while closed habitat species are adapted to lower light conditions with at least a partial canopy. We coded three states for habitat type: (1) close, (2) open, (3) 1 + 2. Life history was scored three states: (1) perennial, (2) annual, and (3) 1 + 2. Ancestral state reconstructions were carried out using the maximum likelihood method in Mesquite v2.75 [45]. Likelihood ratio test was used for model selection between Markov k-state one-parameter (Mk1) and two-parameter (Mk2) models [46]. Mk1 and Mk2 models were identified as optimal for habitat and life history, respectively. The procedure “trace over trees” was used to summarize reconstructions over 1000 randomly sampled BEAST trees (after the burn-in).

Correlation analyses were run using two chronograms obtained in two calibration scenarios, respectively. As range expansion and habitat/life history shifts may influence the diversification of *Oryza*, we firstly tested whether the shift of habitat and/or life history are correlated with intercontinental migrations. We employed the BayesDiscrete method in BayesTraits v2.0 [47] to estimate posterior support for dependent versus independent models of state changes between habitat/life history and range expansion. For each analysis, we performed at least three replicate MCMC runs on the 1000 randomly sampled BEAST trees for 10,000,000 iterations, following a burn-in of 1,000,000 iterations, with posterior sampling every 1000 iterations. The log-Bayes Factor (BF) is calculated as the twice difference between dependent harmonic mean and independent harmonic mean. BF > 2.0 represents positive evidence for correlated evolution between pairs of discrete binary traits [47]. Tropical Asia is the ancestral area of *Oryza* [12, 15], so we coded tropical Asia vs. other regions for geographical ranges. Each species within *Oryza* only has one state for its habitat and life history (Additional file 1: Table S1); thus, we coded close vs. open for habitat and perennial vs. annual for life history.

We further used the binary state speciation and extinction model (BiSSE; [18]) to examine the correlation of habitat and/or life history with the diversification of *Oryza*. To estimate absolute rates of asymmetric character transition (*q*), speciation (λ), and extinction (μ), the ML and Bayesian analyses were conducted in Diversitree v0.4–5 [19]. For a small tree, the ML BiSSE method achieves low power to detect rate asymmetry and estimate model parameters [48]. To increase the power of the ML BiSSE method, we simplified the full model by setting extinction rates to be zero (μ_0_ = μ_1_ = 0) following Davis et al. [48], and then tested the asymmetry of speciation rates under symmetrical transition rates (*q*_01_ = *q*_10_) and asymmetrical (*q*_01_ ≠ *q*_10_). The ΔAIC value between null model (λ_0_ = λ_1_) and alternative model (λ_0_ ≠ λ_1_) and model parameters were then calculated. The distributions of ΔAIC were simulated under the null model. Based on our date estimates, the crown age of *Oryza* was 18.11–24.83 Ma in scenario #1 or 9.36–12.98 Ma in scenario #2, we here sampled 1000 simulated trees with crown age from 9 Ma to 25 Ma and with the same tip state ratio of each trait. The ΔAIC value between null model and alternative model was obtained for each simulated tree. The ΔAIC of *Oryza* data was compared with the distributions of simulated ΔAIC to test significance.

For Bayesian BiSSE analysis, we ran MCMC chains for 10,000 iterations under the model with lowest AIC score. The mean value and credibility intervals of speciation rates were generated after removing 2500 iterations as burn-in. For comparison, we also ran Bayesian BiSSE analysis with the same settings under the 6-parameter full model.

## Results

### Phylogeny and divergence time estimates

**Fig. 2:**
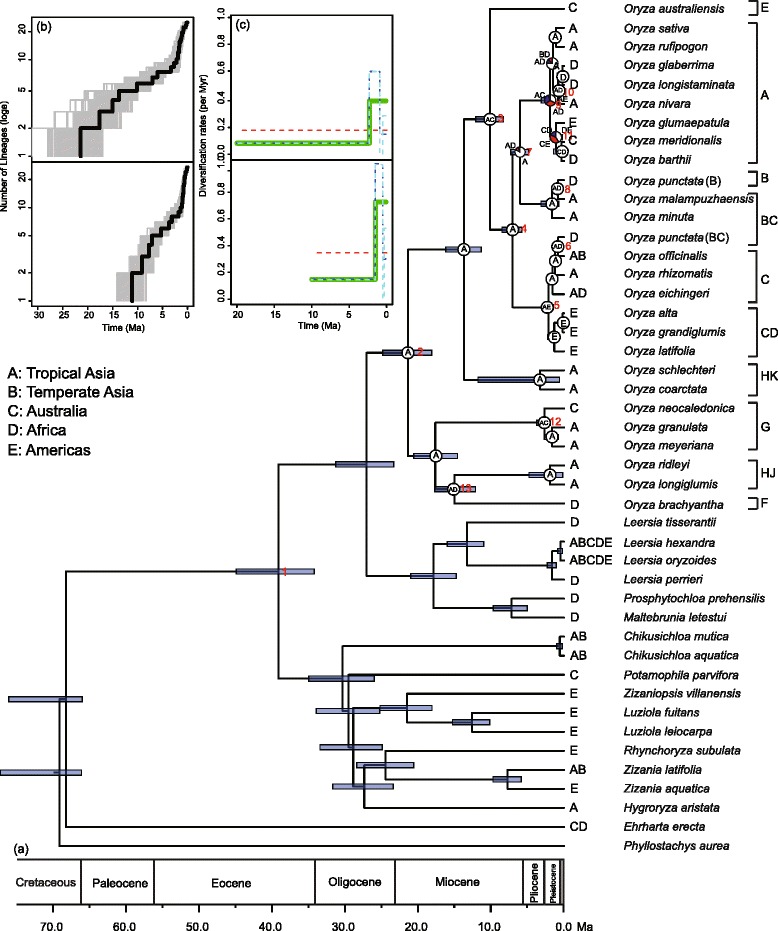
Space and tempo in the evolutionary history of *Oryza*. **a** Combined chronogram and biogeographical analyses of *Oryza*. The MCC tree was generated from the dating analysis of *Oryza* and other Oryzeae in scenario #1. Grey bars represent 95 % highest posterior density intervals for each node. Node charts show the relative probabilities of alternative ancestral distributions obtained by Statistical Dispersal-Vicariance Analysis (S-DIVA) optimisations over the 1000 Bayesian trees (white > red > blue); areas (frequencies < 0.1) are shown in black. Numbers in red near branches indicate the node number, as noted in Table 1. Genome types are shown at the right. **b** Lineage-through-time (LTT) plots for *Oryza*, excluding outgroups. The grey lines represent the results of 1000 trees randomly selected from the BEAST analysis. The black line shows the MCC tree. **c** Maximum-likelihood diversification rate estimates for *Oryza*. The solid line represents the best model and the dashed line represents the other models

Two phylogenetic analyses (BI and ML) of the combined dataset with 20 plastid DNA regions generated a well-supported framework (Additional file 2: Figure S1) that was highly congruent with that produced by BEAST through co-estimation of phylogeny (Fig. 2a). Calibration scenario #1 produced older times than scenario #2. A detailed comparison of estimates of divergence times, based on the two calibration strategies, is shown in Table 1.

**Table 1:** Results for age estimates and biogeographical optimizations with Tropical Asia (A), Temperate Aisa (B), Australia (C) Americas (D) and Africa (E) for the nodes of interest in the Oryzeae

Analysis	Divergence-time estimate (Ma)			Biogeographical inference		
Stem age of Oryzeae	Constrained (66)	Constrained (34.5)	S-DIVA		Lagrange	
Results for discussed nodes	Age (95 % HPD)	Age (95 % HPD)	Ancestral areas	Rel. Prob.	split	Rel. prob.
1 – Crown group of Oryzeae	39.11 (34.23–44.89)	20.34 (17.37–23.35)	–	–	–	–
2 – Crown group of *Oryza*	20.40 (18.11–24.83)	11.10 (9.36–12.98)	A	1.0	A|A	0.50
3 – A-/B-/C-genomes vs. E-genome	10.15 (8.33–12.10)	5.27 (4.31–6.35)	AC	1.0	C|A	0.18
4 – A-/B-genomes vs. C-genome	7.05 (5.80–8.52)	3.68 (2.96–4.44)	A	1.0	A|A	0.25
5 – Migration into South America	2.17 (1.49–2.95)	1.12 (0.79–1.51)	AE	1.0	AD|E	0.34
6 – Migration into Africa	0.81 (0.43–1.24)	0.42 (0.23–0.65)	AD	1.0	D|BA	0.63
7 – A-genome vs. B-genome	6.03 (4.86–7.30)	3.15 (2.51–3.86)	A	0.84	A|A	0.46
			AD	0.14		
8 – Migration into Africa	0.86 (0.49–1.29)	0.46 (0.26–0.70)	AD	1.0	D|A	0.96
9 – Crown group of A-genome	1.91 (1.26–3.14)	1.03 (0.67–1.68)	AE	0.34	A|CDE	0.40
			AD	0.33		
			AC	0.29		
10 – Migration into Africa	0.80 (0.21–1.51)	0.43 (0.09–0.86)	AD	1.0	D|A	0.97
11 – Migration into Africa,	1.14 (0.27–1.91)	0.62 (0.15–1.06)	DE	0.41	E|CD	0.71
Australia, and Americas			CE	0.40		
			CD	0.18		
12 – Migration into Australia	2.71 (1.85–3.74)	1.37 (0.93–1.91)	AC	1.0	C|A	0.90
13 – Migration into Africa	15.02 (12.15–17.70)	7.76 (6.31–9.29)	AD	1.0	A|A	0.50

The node numbers correspond to those in Fig. 1. HPD = highest posterior density intervals; Rel. prob. = relative probability

### Biogeography

Overall, the parsimony-based method revealed results that were very similar to those revealed by the ML-based method under scenarios #1 and #2 (Fig. 2a, Additional file 2: Figure S2; Table 1). Our ancestral area reconstruction suggested a tropical Asian ancestor for *Oryza*. Within *Oryza*, we inferred at least 12 independent dispersal events from tropical Asia into other regions, five dispersals into Africa with speciation events (nodes 6, 8, 9, 10, and 13), two into Americas (nodes 5 and 9), and three into Australia (nodes 3, 9 and 12) (Fig. 2a, Additional file 2: Figure S2).

### Diversification rates

Under two calibration scenarios, the LTT curves of *Oryza* plotted as function of time show that speciation accelerated during the Pleistocene (Fig. 2b). TreePar analyses rejected the null hypothesis of the constant diversification rate of *Oryza* under Yule progress for both scenarios (scenario #1: *χ*^2^ = 14.75, *p* = 0.0006; scenario #2: *χ*^2^ = 14.75, *p* = 0.0006), and found the one-shift model as the best (Fig. 2c). Under scenario #1, *Oryza* began to diversify at a rate of *r*_1_ = 0.083 species/million years (Myr), followed by a shift at 2.2 Ma, increasing to *r*_2_ = 0.399 species/Myr. In scenario #2, the similar results were generated, where a rate shift occurred at 1.4 Ma (*r*_1_ = 0.147 species/Myr, *r*_2_ = 0.725 species/Myr). Under birth-death progress, the TreePar analyses generated similar results under scenarios #1 and #2, respectively.

For BAMM analyses, the results of rate-through-time dynamics indicate that speciation rates increased around 2 Ma under two calibration scenarios (Additional file 2: Figure S4). Estimation of number of regime shifts did not find more than one evolutionary regime of speciation rates under two scenarios (scenario #1: frequency of one regime = 0.38; scenario #2: frequency of one regime = 0.40).

### Correlates of diversification

**Fig. 3:**
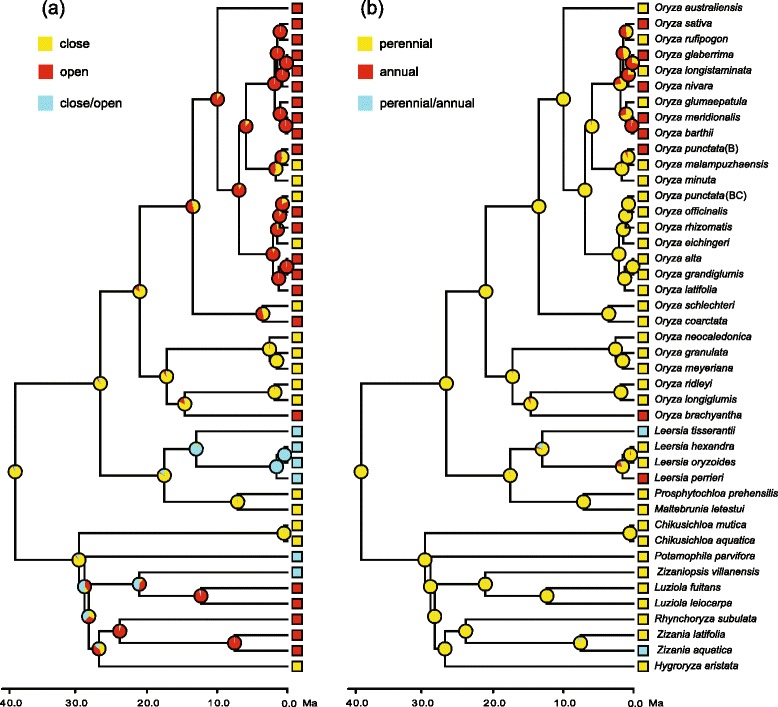
Ancestral state reconstruction in *Oryza* in Mesquite. **a** Habitat. **b** Life history

Our ancestral state reconstructions indicate that close habitat and perennial life history are ancestral states in *Oryza* (Fig. 3). Adaptation to open habitat has evolved independently at least twice; meanwhile, at least three habitat shifts from open to close occurred (Fig. 3a). Annual life history independently originated at least four times; later, two shifts from annual to perennial independently occurred (Fig. 3b).

BayesDiscrete analysis suggested a positive correlation between adaptation to open habitat and dispersal out of tropical Asia (BF = 3.96 > 2.0). However, there was no correlation between annual life history and dispersal out of tropical Asia (BF = 0.06 < 2.0).

**Fig. 4:**
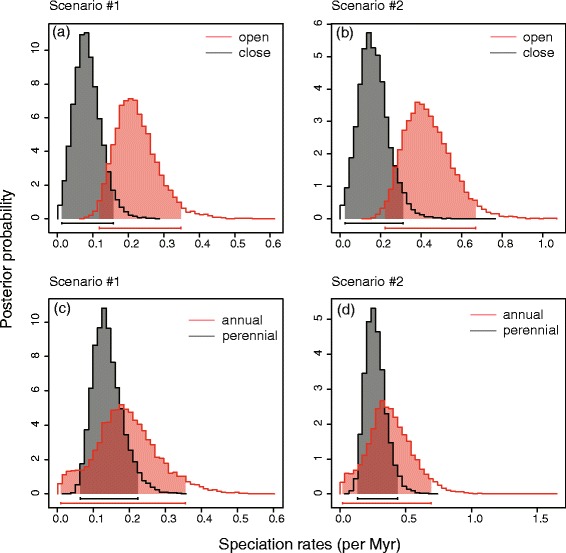
Posterior probability distributions of speciation rates associated with evolutionary changes of habitat (close/open) and life history (perennial/annual) based on Bayesian BiSSE analyses using the model with lowest AIC score under scenario #1 and #2 (see Table 1). The shaded areas and bars indicate the 95 % confidence intervals. **a** Habitat under scenario #1. **b** Habitat under scenario #2. **c** Life history under scenario #1. **d** Life history under scenario #2

The results of ML BiSSE analyses are indicated in Table 2. For habitat, the alternative model (λ_0_ ≠ λ_1_) fits better than the null model (λ_0_ = λ_1_), whether *q*_01_ = *q*_10_ (scenario #1: ΔAIC = −1.71, *p* = 0.02; scenario #2: ΔAIC = −1.68, *p* = 0.03) or not (scenario #1: ΔAIC = −2.62, *p* = 0.04; scenario #2: ΔAIC = −2.53, *p* = 0.04). Speciation rates of close habitat species are lower than of open habitat species, whether *q*_01_ = *q*_10_ or not. For life history, the null model (λ_0_ = λ_1_) fits better when *q*_01_ = *q*_10_ (scenario #1: ΔAIC = 2.17; scenario #2: ΔAIC = 2.32), and the alternative model (λ_0_ ≠ λ_1_) fit better when *q*_01_ ≠ *q*_10_ but the correlation was not significant (scenario #1: ΔAIC = −2.47, *p* = 0.09; scenario #2: ΔAIC = −1.84, *p* = 0.11). The Bayesian analyses of speciation rates generated the similar results for habitat (Fig. 4a, b) and life history (Fig. 4c, d), respectively. The results under the 6-parameter full model are consistent with that under the model with lowest AIC score (Additional file 2: Figure S3).

**Table 2:** Model fit of trait dependent diversification: habitat and life history

Model constraints	λ	lnLik	AIC	ΔAIC
Scenario #1				
Habitat (close/open)				
μ_0_ = μ_1_ = 0	λ_0_ = 0.090, λ_1_ = 0.311	−81.34	170.68	−2.62
λ_0_ = λ_1_, μ_0_ = μ_1_ = 0	λ_0_ = 0.180	−83.65	173.30	
μ_0_ = μ_1_ = 0, *q*_01_ = *q*_10_	λ_0_ = 0.095, λ_1_ = 0.272	−80.82	169.62	−1.71
λ_0_ = λ_1_, μ_0_ = μ_1_ = 0, *q*_01_ = *q*_10_	λ_0_ = 0.180	−82.76	171.33	
Life history (perennial/annual)				
μ_0_ = μ_1_ = 0	λ_0_ = 0.124, λ_1_ = 0.460	−80.12	169.60	−2.47
λ_0_ = λ_1_, μ_0_ = μ_1_ = 0	λ_0_ = 0.180	−82.11	172.07	
μ_0_ = μ_1_ = 0, *q*_01_ = *q*_10_	λ_0_ = 0.144, λ_1_ = 0.522	−85.58	178.98	2.17
λ_0_ = λ_1_, μ_0_ = μ_1_ = 0, *q*_01_ = *q*_10_	λ_0_ = 0.180	−85.46	176.81	
Scenario #2				
Habitat (close/open)				
μ_0_ = μ_1_ = 0	λ_0_ = 0.176, λ_1_ = 0.591	−66.23	138.12	−2.53
λ_0_ = λ_1_, μ_0_ = μ_1_ = 0	λ_0_ = 0.346	−68.52	140.65	
μ_0_ = μ_1_ = 0, *q*_01_ = *q*_10_	λ_0_ = 0.185, λ_1_ = 0.521	−66.69	137.00	−1.68
λ_0_ = λ_1_, μ_0_ = μ_1_ = 0, *q*_01_ = *q*_10_	λ_0_ = 0.346	−68.54	138.68	
Life history (perennial/annual)				
μ_0_ = μ_1_ = 0	λ_0_ = 0.247, λ_1_ = 0.843	−65.85	137.50	−1.84
λ_0_ = λ_1_, μ_0_ = μ_1_ = 0	λ_0_ = 0.346	−67.81	139.34	
μ_0_ = μ_1_ = 0, *q*_01_ = *q*_10_	λ_0_ = 0.277, λ_1_ = 0.965	−71.30	146.26	2.32
λ_0_ = λ_1_, μ_0_ = μ_1_ = 0, *q*_01_ = *q*_10_	λ_0_ = 0.346	−71.14	143.94	

Four models with different constraint settings on speciation rates (λ), extinction rates (μ), and state-transition rates (*q*) were implied. Subscript numbers 0 and 1 refer to close/open habitats and perennial/annual life histories, respectively. Constraint λ_0_ = λ_1_ assumes that the speciation rates of lineages with trait 0 and trait 1 are equal, μ_0_ = μ_1_ = 0 assumes that the extinction rates of lineages with trait 0 and trait 1 are zero, and q_01_ = q_10_ assumes that transition rates from trait 0 to trait 1 and from trait 1 to trait 0 are equal

## Discussion

### Phylogeny and diversification of *Oryza*

Based on the combined twenty-marker DNA dataset, genus-level relationships within of Oryzeae were well resolved, which are in agreement with previous studies [12, 49]. The phylogenetic relationships among diploid species are congruent with the results obtained by nuclear data [10, 11]. The comparison between the calibration scenarios (Table 1) suggests that phytolith fossils from the late Cretaceous as a divergent calibration point strongly affect divergence time estimations for deep nodes in Oryzeae and *Oryza*, which is in agreement with recent molecular dating studies on Poaceae [25, 49]. In scenario #1, the stem ages of Oryzeae and *Oryza* are highly congruent with the results of previous molecular dating analyses [25, 49]. Our estimations for *Oryza* in scenario #2 are highly congruent with the previous study [12]. This is not surprised because the similar calibration points were used though using different analytical methods.

Within *Oryza*, two early rapid diversification events were hypothesized based on divergence time estimates [13], involving two and three extant lineages, respectively. Our LTT plots and TreePar analyses in scenario #1 and #2 generated the similar results and only detected a significant increase in diversification rates around 2 Ma (Fig. 2b, c). It is possible that LTT plots and TreePar analyses may be not sensitive to rapid speciation events involving only a few lineages. The rate-through-time dynamics of BAMM analyses also suggested an elevated diversification rate around 2 Ma (Additional file 2: Figure S4), while one evolutionary regime was selected by the estimation of number of regime shifts in BAMM. The BAMM is commonly applied to broadly sampled trees, such as root age of tens or hundreds of millions of years and family-level sampling [50–52], and its power to detect rate variation decreases in small trees [50, 53].

### Diversification mechanisms of *Oryza*

Our results indicate that the most recent common ancestor of the rice genus occurred in tropical Asia at 20.40 Ma (95 % HPD: 18.11–24.83) or 11.10 Ma (95 % HPD: 9.36–12.98). The current biogeographical pattern of the genus primarily resulted from recent intercontinental dispersals (Fig. 2a, Additional file 2: Figure S2). The Asian origin of *Oryza* and its long-distance dispersals to other continents have also been proposed by previous studies [12, 14–16]. According to our analysis, at least 12 migration events in *Oryza* occurred from tropical Asia into other regions (Fig. 2a, Additional file 2: Figure S2). Given the inferred credibility intervals of the estimated times of divergence, the majority of the dispersal events (10/12) have occurred since the Pleistocene. These were 8 of 10 dispersal events with speciation (Table 1). In Africa, three were associated with anagenesis (when the colonist species leads to only one species) and one was associated with cladogenesis (when a single colonization event gives rise to at least two species). In Americas, one was associated with anagenesis and one with cladogenesis (resulting to three species). In Australia, two were associated with anagenesis. During the Pleistocene, dispersals had a greater contribution than *in situ* speciation (8 vs. 4). Our diversification analyses also indicate that *Oryza* underwent a rapid diversification during the same period (Fig. 2b, c, Additional file 2: Figure S4). Thus, we suggest that the range expansions from tropical Asia to other regions are responsible for the rapid diversification of the genus during the Pleistocene.

The increased rates of diversification in a group may be attributed to the interplay between opportunity and key innovation (e.g. [54, 55]). Here, we tested whether transitions within two ecological traits, habitat and life history, caused the increase in diversification rates of *Oryza* during the Pleistocene. Our ancestral state reconstruction indicates that adaptation to open habitat was derived in *Oryza* (Fig. 3a). Within the genus, 17 of 26 species (~63 %) inhabit open habitat (Additional file 1: Table S1). BiSSE analyses indicate asymmetry in speciation rates between close and open habitat species, and open habitat species had higher speciation rates (Fig. 4a, b, Additional file 2: Figure S3a, c; Table 2). BayesDiscrete analysis suggests that open habitat species are more likely to migrate from tropical Asia to other regions.

Adaptive radiation theory predicts that a shift to a new habitat may provide new ecological opportunities to lineages, leading to increased rates of diversification [2, 56]. After sudden stepped cooling events occurred during the Pliocene–Pleistocene transition (~2.7 Ma), driven predominantly by declining atmospheric *p*CO_2_ levels [57], there was an intermittent transient warm period until ~2.2 Ma, after which the global climate descended into glacial conditions [58]. Based on sea-level reconstruction, Rohling *et al.* [59] recently identified the first major glaciation at 2.15 Ma. The climate became drier after the Pliocene–Pleistocene transition [60]. Within *Oryza*, the majority of open habitat species inhabit seasonally dry habitats, and species adapting close habitat usually inhabit forests (Additional file 1: Table S1). Thus, open habitat species may have performed better than close habitat species in the drier and cooler environments that characterised the Pleistocene.

Within *Oryza*, two independent transitions from close to open habitats occurred during the Miocene (Fig. 3a). Significantly, 15 of 18 species (88.2 %) within the A-/B-/C-/E-genome lineages are species adapting open habitat. They began to differentiate at 10.15 Ma (95 % HPD: 8.33–12.10) or 5.27 Ma (95 % HPD: 4.31–6.35) based on different calibration strategies, markedly earlier than the increased rate of diversification (ca. 2 Ma). Bayesian MCMC analysis supports a positive correlation between open habitat and dispersal out of tropical Asia (BF > 2). The majority of the migration events (10/12) seem to have happened during the Pleistocene. Therefore, rice species may have been pre-adapted to invade open habitat. Thus, we propose that range expansion and invasion of novel habitats (here seen as pre-adaptation) are principal factors resulting in the observed pattern of increased diversification in *Oryza*.

Some studies have indicated that life history shifts are responsible for increased rates of diversification of many groups, such as *Lupinus* [55] and the phaseoloid legumes [61]. Our ancestral state reconstruction suggests that perennial life history is the ancestral state in *Oryza*, whereas annual life history has independently evolved at least four times (Fig. 3b). The results from BiSSE analyses did not show a significant difference in differential rates of diversification between annual and perennial species (Fig. 4c, d, Additional file 2: Figure S3e, g; Table 2). Type 2 errors may have occurred due to the small size of the tree of *Oryza* [48]. However, Bayesian MCMC analysis did not positively support a correlation between range expansion and life history shifts (BF < 2).

According to life history theory, annual species of plants maybe outcomplete perennials in dry environments with high seasonality fluctuation in water availability [62, 63]. Annual species are generally favoured in warmer and drier climates, whereas perennial species are favoured in cooler and wetter environments (e.g. [55, 64, 65]. Within *Oryza*, seven species are annuals (Additional file 1: Table S1). Their habitats are seasonally dry [9], but they grow in ponds and swamps, near streams, or in water in seasonally inundated areas and therefore are not constrained by water availability. In fact, the habitats of annual species within the rice genus do not differ from those of their perennial relatives (see Additional file 1: Table S1 for more details). Thus, life history shift may not independently drive the increased diversification of rice species without habitat differentiations.

## Conclusions

By assembling a twenty-locus plastid dataset, we present a phylogeny for all species of *Oryza*. Our analyses show that *Oryza* became differentiated in tropical Asia in the Miocene, but increased diversification did not occurred until the Pleistocene. We inferred at least 10 dispersal events out of tropical Asia since the Pleistocene, mainly involving open habitat species. Intercontinental dispersals are positively correlated with habitat shift from close to open, but are not correlated with life history shift from perennial to annual. The speciation rate in open habitat is higher than that in close habitat, whereas the speciation rate of annual species does not differ from that of perennial species. Thus, range expansion triggered increased diversification of *Oryza* during the Pleistocene together with habitat shift, not with life history shift. However, the invasion of open habitat predated the burst in diversification rates, and accordingly is a pre-adaptation, not a key innovation. These results illustrate that, for *Oryza* and perhaps other lineages of organisms, range expansion and invasion of novel habitats may be principal factors resulting in increased diversification.

## Additional files

Additional file 1: Table S1 and Table S2.
**Table S1:** Species of *Oryza*, including their genome types, life histories, habitats, and geographic distributions. **Table S2:** Species and their GenBank accession numbers used in this study. (PDF 42 kb) Additional file 2: Figure S1 and Table S4.
**Table S1:** ML tree of *Oryza*. **Figure S2:** Biogeographical inference for *Oryza* based on Lagrange. **Figure S3:** Posterior probability distributions for the speciation and extinction rates obtained from Bayesian BiSSE using 6-paramter full model. **Figure S4:** Rate-through-time dynamics for *Oryza* inferred by BAMM. (PDF 265 kb)
