# Supplementary material for: Range expansion and habitat shift triggered elevated diversification of the rice genus (Oryza, Poaceae) during the Pleistocene
Source: BMC Evol Biol. 2015 Sep 3;15:182. doi: 10.1186/s12862-015-0459-1 (PMC4559288; doi:10.1186/s12862-015-0459-1)
Supplement: Additional file 1: Table S1 and Table S2. — Table S1: Species of Oryza, including their genome types, life histories, habitats, and geographic distributions. Table S2: Species and their GenBank accession numbers used in this study. (PDF 42 kb) [file 12862_2015_459_MOESM1_ESM.pdf]

Additional file 1

Table S1. Species of *Oryza*, including their genome types, life histories, habitats, and geographical distributions.

Table S2. GenBank accession numbers for the species used in this study.

Table S1

Species	Genome	Life history	Habitat	Distribution
Oryza alta	CCDD	Perennial	Open (seasonally wet places or water in savanna and woodland)	South and Central America
*Oryza australiensis**	EE	Perennial-biennial	Open (seasonally wet places, such as swamps, the edges of freshwater lagoons, seasonally dry pools, alluvial streams, or behind river levees)	Tropical Australia
Oryza barthii	AA	Annual	Open (seasonally flood land, stagnant water, and slowly flowing water of pools)	Sub-Saharan Africa
Oryza brachyantha	FF	Annual	Open (ponds, near streams, seasonally inundated areas in water)	Sub-Saharan Africa
Oryza coarctata	HHKK	Perennial	Open (brackish water the coastal and tidal regions)	India and Myanmar
Oryza eichingeri	CC	Perennial	Close (damp or flooded sites in forest)	South Asia, East Africa
Oryza glaberrima	AA	Annual	Open (upland to deepwater)	West Africa
Oryza glumaepatula	AA	Perennial	Open (flood plains that become seasonally dry)	South and Central America
Oryza grandiglumis	CCDD	Perennial	Open (seasonally wet places or water in savanna and woodland)	South and Central America
Oryza granulata	GG	Perennial	Close (damp places in forest, including beside streamsides, near waterfalls or water holes, or seasonally dry sloping land)	South and Southeast Asia
Oryza latifolia	CCDD	Perennial	Open (seasonally wet places in forest, woodland, savanna, pasture, cultivated fields,	South and Central America
Oryza longiglumis	HHJJ	Perennial	Close (seasonally wet areas such as swamps, water holes of stream beds in forest)	Indonesia, Papua New Guinea
Oryza longistaminata	AA	Perennial	Open (seasonally dry to deepwater)	Sub-Saharan Africa
Oryza malampuzhaensis	BBCC	Perennial	Close (seasonally dry forest pools)	South India
*Oryza meridionalis**	AA	Annual-biennial	Open (the edges of seasonally freshwater lagoons, temporary pools, and swamps)	Tropical Australia
Oryza meyeriana	GG	Perennial	Close (streams or dry riverbanks in forest)	Southeast Asia
Oryza minuta	BBCC	Perennial	Close (streamsides and riverbanks)	Philippines, Papua New Guinea
Oryza neocaledonica	GG	Perennial	Close (dry forests)	New Caledonian
*Oryza nivara**	AA	Annual-biennial	Open (seasonally wet places and shallow water)	(Sub)tropical Asia
Oryza officinalis	CC	Perennial	Open (seasonally wet places, ditches, swampy/marshy places, near small water holes, and along lakesides, streams, or rivers)	(Sub)tropical Asia
Oryza puncatata (B)	BB	Annual	Open (seasonally swamp areas, around water holes and pools, on riverbanks in areas that flood to 1 m depth)	Sub-Saharan Africa
Oryza puncatata (BC)	BBCC	Perennial	Close (wet places in forest)	Sub-Saharan Africa
Oryza rhizomatis	CC	Perennial	Open (seasonally swampy or periodically flooded areas in forest and scrub)	Sri Lanka
Oryza ridleyi	HHJJ	Perennial	Close (seasonally wet areas such as marshes or near streamsides in forest)	Southeast Asia
Oryza rufipogon	AA	Perennial	Open (seasonally deepwater and wet year round)	(Sub)tropical Asia
*Oryza sativa**	AA	Annual-biennial	Open (upland to deepwater)	Tropical Asia
Oryza schlechteri	HHKK	Perennial	Close (wet places in forest)	Indonesia, Papua New Guinea

Species with a star are sometimes described as different life histories when they occur

in different populations or regions. Three species, *O. meridionalis*, *O. nivara*, and *O. sativa*, are described as annual or biennial. In this study, we coded them as annual. *O. australiensis* is described as perennial or biennial; we coded it as perennial.

Table S2

	GenBank Accession																			
Species	*atpB-rbcL*	*atpF*	*atpI-atpH*	*rps16-trnQ*	*matK*	*ndhA*	*ndhC-trnV*	*ndhF*	*petB*	*rpl20-clpP*	*rps3-rps19*	*rps16*	*trnC-rpoB*	*trnG*	*trnL*	*trnP-rpl33*	*trnS-trnfM*	*trnT-trnD*	*trnV*	*ycf3*
Chikusichloa aquatica	FJ908118	FJ908151	FJ908217	FJ908250	AF489912	FJ908292	FJ908324	FJ908357	FJ908390	FJ908184	FJ908456	FJ908423	FJ908489	FJ908522	AY792531	FJ908564	FJ908597	FJ908630	FJ908662	FJ908695
Chikusichloa mutica	FJ908119	FJ908152	FJ908218	FJ908251	FJ908269	FJ908293	FJ908325	FJ908358	FJ908391	FJ908185	FJ908457	FJ908424	FJ908490	FJ908523	FJ908541	FJ908565	FJ908598	FJ908631	FJ908663	FJ908696
Ehrharta erecta	FJ908127	FJ908160	FJ908226	FJ908259	AY792568	FJ908301	FJ908333	FJ908366	FJ908399	FJ908193	FJ908465	FJ908432	FJ908498	FJ908531	AY792539	FJ908573	FJ908606	FJ908639	FJ908671	FJ908704
Hygroryza aristata	FJ908126	FJ908159	FJ908225	FJ908258	AF489913	FJ908300	FJ908332	FJ908365	FJ908398	FJ908192	FJ908464	FJ908431	FJ908497	FJ908530	AY792538	FJ908572	FJ908605	FJ908638	FJ908670	FJ908703
Leersia hexandra	FJ908113	FJ908146	FJ908212	FJ908245	AF489909	FJ908287	FJ908320	FJ908352	FJ908385	FJ908179	FJ908451	FJ908418	FJ908484	FJ908517	AY792527	FJ908559	FJ908592	FJ908625	FJ908658	FJ908690
Leersia oryzoides	FJ908111	FJ908144	FJ908210	FJ908243	AY792566	FJ908285	FJ908318	FJ908350	FJ908383	FJ908177	FJ908449	FJ908416	FJ908482	FJ908515	AY792525	FJ908557	FJ908590	FJ908623	FJ908656	FJ908688
Leersia perrieri	FJ908112	FJ908145	FJ908211	FJ908244	AF148677	FJ908286	FJ908319	FJ908351	FJ908384	FJ908178	FJ908450	FJ908417	FJ908483	FJ908516	AY792526	FJ908558	FJ908591	FJ908624	FJ908657	FJ908689
Leersia tisserantii	FJ908114	FJ908147	FJ908213	FJ908246	AF489910	FJ908288	FJ908321	FJ908353	FJ908386	FJ908180	FJ908452	FJ908419	FJ908485	FJ908518	AY792528	FJ908560	FJ908593	FJ908626	FJ908659	FJ908691
Luziola fluitans	FJ908123	FJ908156	FJ908222	FJ908255	AY792567	FJ908297	FJ908329	FJ908362	FJ908395	FJ908189	FJ908461	FJ908428	FJ908494	FJ908527	AY792537	FJ908569	FJ908602	FJ908635	FJ908667	FJ908700
Luziola leiocarpa	FJ908124	FJ908157	FJ908223	FJ908256	AF489911	FJ908298	FJ908330	FJ908363	FJ908396	FJ908190	FJ908462	FJ908429	FJ908495	FJ908528	AY792536	FJ908570	FJ908603	FJ908636	FJ908668	FJ908701
Maltebrunia letestui	FJ908115	FJ908148	FJ908214	FJ908247	FJ908267	FJ908289		FJ908354	FJ908387	FJ908181	FJ908453	FJ908420	FJ908486	FJ908519	FJ908539	FJ908561	FJ908594	FJ908627		FJ908692
Oryza alta					AF148664		AB436262								GU595104					
Oryza australiensis	FJ908105	FJ908138	FJ908204	FJ908237	AF148667	FJ908279	FJ908312	FJ908344	FJ908377	FJ908171	FJ908443	FJ908410	FJ908476	FJ908509	AY792521	FJ908551	FJ908584	FJ908617	FJ908650	FJ908682
Oryza barthii					AF148655		AB436245													
Oryza brachyantha	FJ908107	FJ908140	FJ908206	FJ908239	AF148670	FJ908281	FJ908314	FJ908346	FJ908379	FJ908173	FJ908445	FJ908412	FJ908478	FJ908511	AY792523	FJ908553	FJ908586	FJ908619	FJ908652	FJ908684
Oryza coarctata	FJ908106	FJ908139	FJ908205	FJ908238	AF148669	FJ908280	FJ908313	FJ908345	FJ908378	FJ908172	FJ908444	FJ908411	FJ908477	FJ908510	AY792522	FJ908552	FJ908585	FJ908618	FJ908651	FJ908683
Oryza eichingeri					AF148659		AB436252								GU595108					
Oryza glaberrima	FJ908097	FJ908130	FJ908196	FJ908229	AF148654	FJ908271	FJ908304	FJ908336	FJ908369	FJ908163	FJ908435	FJ908402	FJ908468	FJ908501	AY749371	FJ908543	FJ908576	FJ908609	FJ908642	FJ908674
Oryza glumaepatula					AF148653		AB436235													
Oryza grandiglumis					AF148666		AB436265													
Oryza granulata	FJ908109	FJ908142	FJ908208	FJ908241	AF148674	FJ908283	FJ908316	FJ908348	FJ908381	FJ908175	FJ908447	FJ908414	FJ908480	FJ908513	AY792524	FJ908555	FJ908588	FJ908621	FJ908654	FJ908686
Oryza latifolia	FJ908104	FJ908137	FJ908203	FJ908236	FJ908264	FJ908278	FJ908311	FJ908343	FJ908376	FJ908170	FJ908442	FJ908409	FJ908475	FJ908508	FJ908536	FJ908550	FJ908583	FJ908616	FJ908649	FJ908681
Oryza longiglumis					AF148672		AB436272													
Oryza longistaminata					AY768779		AB436240													
Oryza malampuzhaensis	FJ908100	FJ908133	FJ908199	FJ908232	FJ908262	FJ908274	FJ908307	FJ908339	FJ908372	FJ908166	FJ908438	FJ908405	FJ908471	FJ908504	FJ908534	FJ908546	FJ908579	FJ908612	FJ908645	FJ908677

Oryza meridionalis	FJ908098	FJ908131	FJ908197	FJ908230	AF148657	FJ908272	FJ908305	FJ908337	FJ908370	FJ908164	FJ908436	FJ908403	FJ908469	FJ908502	AY749374	FJ908544	FJ908577	FJ908610	FJ908643	FJ908675
Oryza meyeriana					AF148673		AB436280													
Oryza minuta					AF148663		AB436250													
Oryza neocaledonica	FJ908110	FJ908143	FJ908209	FJ908242	FJ908266	FJ908284	FJ908317	FJ908349	FJ908382	FJ908176	FJ908448	FJ908415	FJ908481	FJ908589	FJ908538	FJ908556		FJ908622	FJ908655	FJ908687
Oryza nivara					AF148652		AP006728													
Oryza officinalis	FJ908101	FJ908134	FJ908200	FJ908233	AF148658	FJ908275	FJ908308	FJ908340	FJ908373	FJ908167	FJ908439	FJ908406	FJ908472	FJ908505	AY792519	FJ908547	FJ908580	FJ908613	FJ908646	FJ908678
Oryza punctata (B)	FJ908099	FJ908132	FJ908198	FJ908231	AF148661	FJ908273	FJ908306	FJ908338	FJ908371	FJ908165	FJ908437	FJ908404	FJ908470	FJ908503	AY792518	FJ908545	FJ908578	FJ908611	FJ908644	FJ908676
Oryza punctata (BC)	FJ908102	FJ908135	FJ908201	FJ908234	FJ908263	FJ908276	FJ908309	FJ908341	FJ908374	FJ908168	FJ908440	FJ908407	FJ908473	FJ908506	FJ908535	FJ908548	FJ908581	FJ908614	FJ908647	FJ908679
Oryza rhizomatis	FJ908103	FJ908136	FJ908202	FJ908235	AF148660	FJ908277	FJ908310	FJ908342	FJ908375	FJ908169	FJ908441	FJ908408	FJ908474	FJ908507	AY792520	FJ908549	FJ908582	FJ908615	FJ908648	FJ908680
Oryza ridleyi	FJ908108	FJ908141	FJ908207	FJ908240	FJ908265	FJ908282	FJ908315	FJ908347	FJ908380	FJ908174	FJ908446	FJ908413	FJ908479	FJ908512	FJ908537	FJ908554	FJ908587	FJ908620	FJ908653	FJ908685
Oryza rufipogon	FJ908096	FJ908129	FJ908195	FJ908228	FJ908261	FJ908270	FJ908303	FJ908335	FJ908368	FJ908162	FJ908434	FJ908401	FJ908467	FJ908500	FJ908533	FJ908542	FJ908575	FJ908608	FJ908641	FJ908673
Oryza schlechteri					AF148668															
Oyrza sativa	X15901	X15901	X15901	X15901	X15901	X15901	X15901	X15901	X15901	X15901	X15901	X15901	X15901	X15901	X15901	X15901	X15901	X15901	X15901	X15901
Phyllostachys aurea	FJ908128	FJ908161	FJ908227	FJ908260	AF164390	FJ908302	FJ908334	FJ908367	FJ908400	FJ908194	FJ908466	FJ908433	FJ908499	FJ908532	AY792540	FJ908574	FJ908607	FJ908640	FJ908672	FJ908705
Potamophila parviflora	FJ908117	FJ908150	FJ908216	FJ908249	AF489916	FJ908291	FJ908323	FJ908356	FJ908389	FJ908183	FJ908455	FJ908422	FJ908488	FJ908521	AY792530	FJ908563	FJ908596	FJ908629	FJ908661	FJ908694
Prosphytochloa prehensilis	FJ908116	FJ908149	FJ908215	FJ908248	FJ908268	FJ908290	FJ908322	FJ908355	FJ908388	FJ908182	FJ908454	FJ908421	FJ908487	FJ908520	FJ908540	FJ908562	FJ908595	FJ908628	FJ908660	FJ908693
Rhynchoryza subulata	FJ908122	FJ908155	FJ908221	FJ908254	AF148675	FJ908296	FJ908328	FJ908361	FJ908394	FJ908188	FJ908460	FJ908427	FJ908493	FJ908526	AY792532	FJ908568	FJ908601	FJ908634	FJ908666	FJ908699
Zizania aquatica	FJ908120	FJ908153	FJ908219	FJ908252	AF164393	FJ908294	FJ908326	FJ908359	FJ908392	FJ908186	FJ908458	FJ908425	FJ908491	FJ908524	AY792533	FJ908566	FJ908599	FJ908632	FJ908664	FJ908697
Zizania latifolia	FJ908121	FJ908154	FJ908220	FJ908253	AY092064	FJ908295	FJ908327	FJ908360	FJ908393	FJ908187	FJ908459	FJ908426	FJ908492	FJ908525	AY792534	FJ908567	FJ908600	FJ908633	FJ908665	FJ908698
Zizaniopsis villanensis	FJ908125	FJ908158	FJ908224	FJ908257	AF148676	FJ908299	FJ908331	FJ908364	FJ908397	FJ908191	FJ908463	FJ908430	FJ908496	FJ908529	AY792535	FJ908571	FJ908604	FJ908637	FJ908669	FJ908702
