# Supplementary material for: Range expansion and habitat shift triggered elevated diversification of the rice genus (Oryza, Poaceae) during the Pleistocene
Source: BMC Evol Biol. 2015 Sep 3;15:182. doi: 10.1186/s12862-015-0459-1 (PMC4559288; doi:10.1186/s12862-015-0459-1)
Supplement: Additional file 2: Figure S1 and Table S4. — Table S1: ML tree of Oryza. Figure S2: Biogeographical inference for Oryza based on Lagrange. Figure S3: Posterior probability distributions for the speciation and extinction rates obtained from Bayesian BiSSE using 6-paramter full model. Figure S4: Rate-through-time dynamics for Oryza inferred by BAMM. (PDF 265 kb) [file 12862_2015_459_MOESM2_ESM.pdf]

Additional file 2

Figure S1. ML tree of *Oryza*. Numbers above and below branches are bootstrap percentages and Bayesian posterior probabilities, respectively. Numbers in the black dots indicate calibration points used in this study.

Figure S2. Biogeographical inference for *Oryza* based on Lagrange. The MCC tree was generated from the dating analysis of *Oryza* and other Oryzeae in scenario #1. The results in scenario #2 were same in each node of the MCC tree. The letters above and below branches indicate the splits estimated by Lagrange. The numbers beside the node indicate the relative probability of the optimal reconstructions.

Figure S3. Posterior probability distributions for the speciation and extinction rates obtained from Bayesian BiSSE using 6-paramter full model. The shaded areas and bars indicate the 95% confidence intervals. (a) and (b) Close vs. open habitat lineages under scenario #1. (c) and (d) Close vs. open habitat lineages under scenario #2. (e) and (f) Perennial vs. annual lineages under scenario #1. (g) and (h) Perennial vs. annual lineages under scenario #2.

Figure S4. Rate-through-time dynamics for *Oryza* inferred by BAMM. The red line represents the mean rates and the blue lines represent the 95% confidence interval.

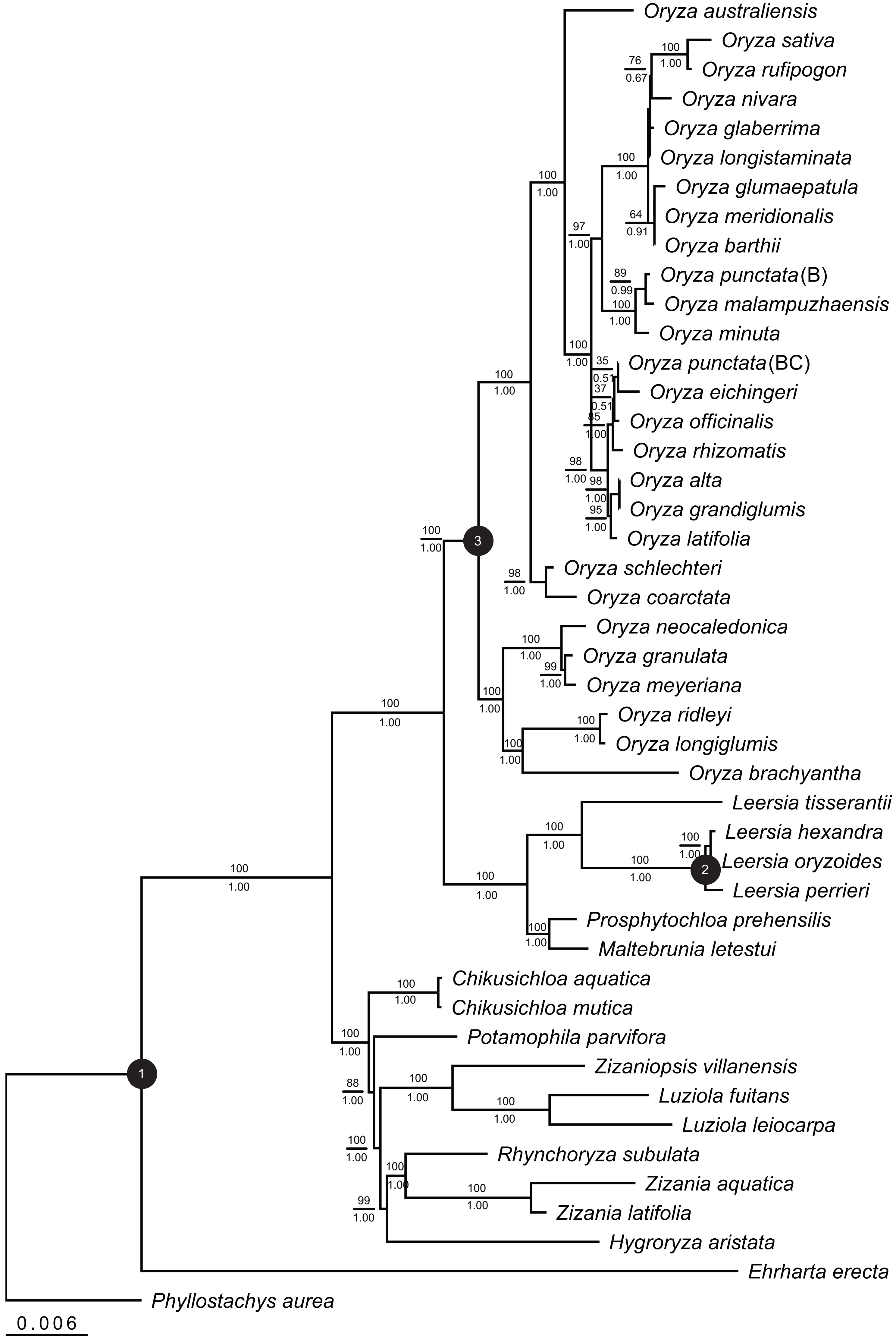

Oryza australiensis
Oryza sativa
Oryza rufipogon
Oryza nivara
Oryza glaberrima
Oryza longistaminata
Oryza glumaepatula
Oryza meridionalis
Oryza barthii
Oryza punctata (B)
Oryza malampuzhaensis
Oryza minuta
Oryza punctata (BC)
Oryza eichingeri
Oryza officinalis
Oryza rhizomatis
Oryza alta
Oryza grandiglumis
Oryza latifolia
Oryza schlechteri
Oryza coarctata
Oryza neocaledonica
Oryza granulata
Oryza meyeriana
Oryza ridleyi
Oryza longiglumis
Oryza brachyantha
Leersia tisserantii
Leersia hexandra
Leersia oryzoides
Leersia perrieri
Prosphytochloa prehensilis
Maltebrunia letestui
Chikusichloa aquatica
Chikusichloa mutica
Potamophila parvifora
Zizaniopsis villanensis
Luziola fuitans
Luziola leiocarpa
Rhynchoryza subulata
Zizania aquatica
Zizania latifolia
Hygroryza aristata
Ehrharta erecta
Phyllostachys aurea
0.006
1
2
3

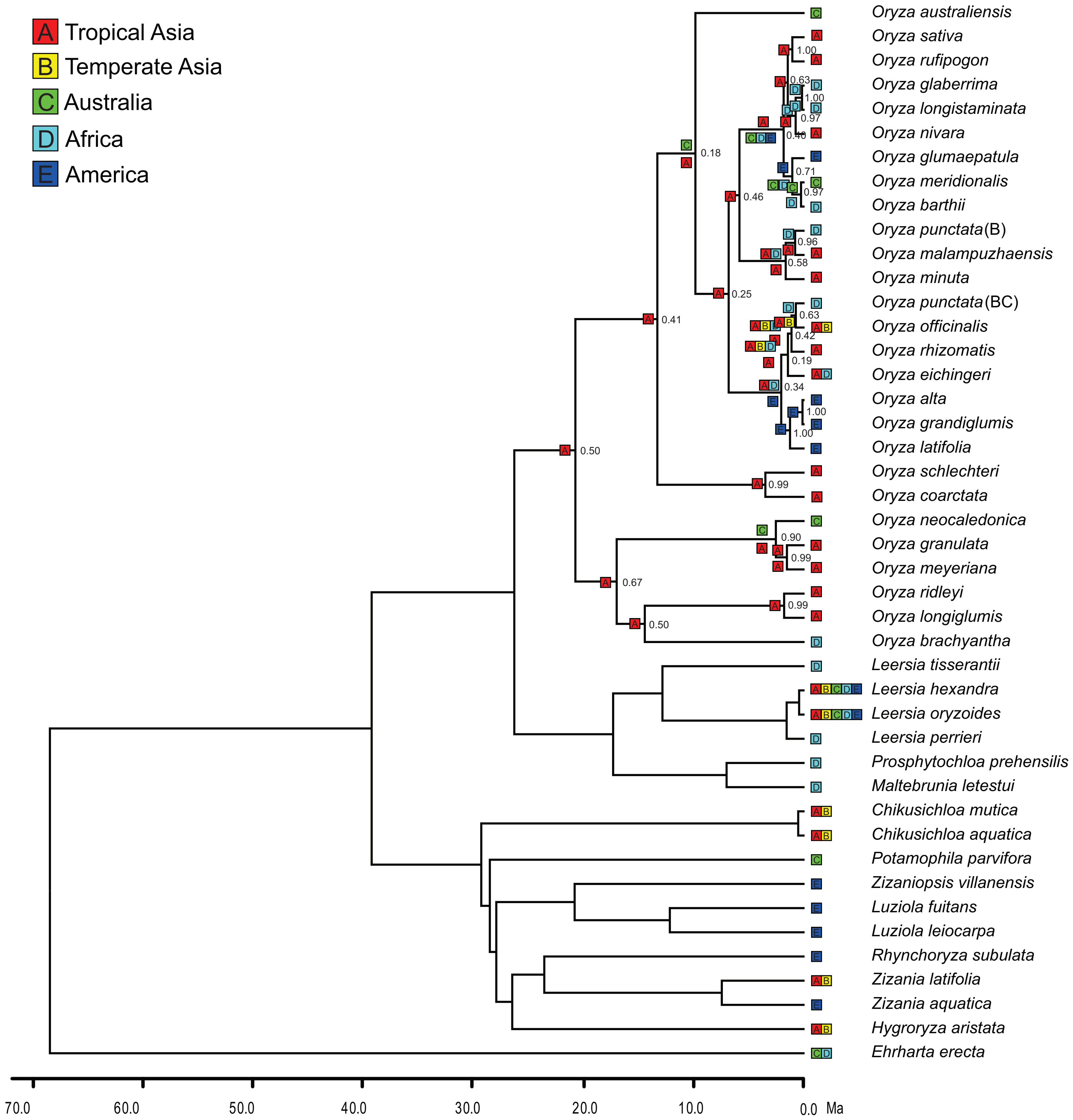

A Tropical Asia
B Temperate Asia
C Australia
D Africa
E America
Oryza australiensis
Oryza sativa
Oryza rufipogon
Oryza glaberrima
Oryza longistaminata
Oryza nivara
Oryza glumaepatula
Oryza meridionalis
Oryza barthii
Oryza punctata(B)
Oryza malampuzhaensis
Oryza minuta
Oryza punctata(BC)
Oryza officinalis
Oryza rhizomatis
Oryza eichingeri
Oryza alta
Oryza grandiglumis
Oryza latifolia
Oryza schlechteri
Oryza coarctata
Oryza neocaledonica
Oryza granulata
Oryza meyeriana
Oryza ridleyi
Oryza longiglumis
Oryza brachyantha
Leersia tisserantii
Leersia hexandra
Leersia oryzoides
Leersia perrieri
Prosphytochloa prehensilis
Maltebrunia letestui
Chikusichloa mutica
Chikusichloa aquatica
Potamophila parvifora
Zizaniopsis villanensis
Luziola fuitans
Luziola leiocarpa
Rhynchoryza subulata
Zizania latifolia
Zizania aquatica
Hygroryza aristata
Ehrharta erecta
70.0
60.0
50.0
40.0
30.0
20.0
10.0
0.0 Ma

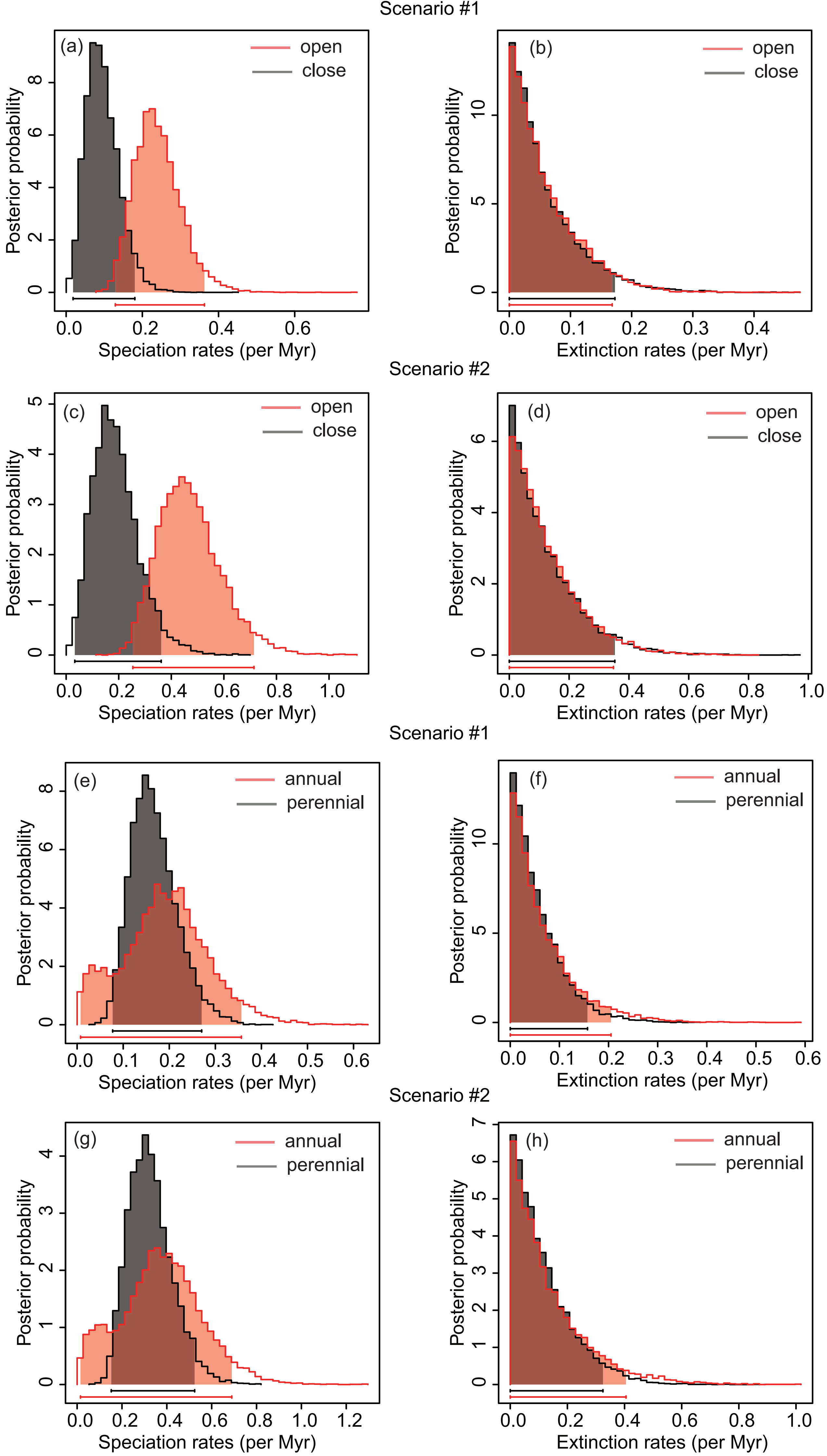

Scenario #1
(a)
open
close
Posterior probability
0
2
4
6
8
0.0
0.2
0.4
0.6
Speciation rates (per Myr)
(b)
open
close
Posterior probability
0
5
10
0.0
0.1
0.2
0.3
0.4
Extinction rates (per Myr)
Scenario #2
(c)
open
close
Posterior probability
0
1
2
3
4
5
0.0
0.2
0.4
0.6
0.8
1.0
Speciation rates (per Myr)
(d)
open
close
Posterior probability
0
2
4
6
0.0
0.2
0.4
0.6
0.8
1.0
Extinction rates (per Myr)
Scenario #1
(e)
annual
perennial
Posterior probability
0
2
4
6
8
0.0
0.1
0.2
0.3
0.4
0.5
0.6
Speciation rates (per Myr)
(f)
annual
perennial
Posterior probability
0
5
10
0.0
0.1
0.2
0.3
0.4
0.5
0.6
Extinction rates (per Myr)
Scenario #2
(g)
annual
perennial
Posterior probability
0
1
2
3
4
0.0
0.2
0.4
0.6
0.8
1.0
1.2
Speciation rates (per Myr)
(h)
annual
perennial
Posterior probability
0
1
2
3
4
5
6
7
0.0
0.2
0.4
0.6
0.8
1.0
Extinction rates (per Myr)

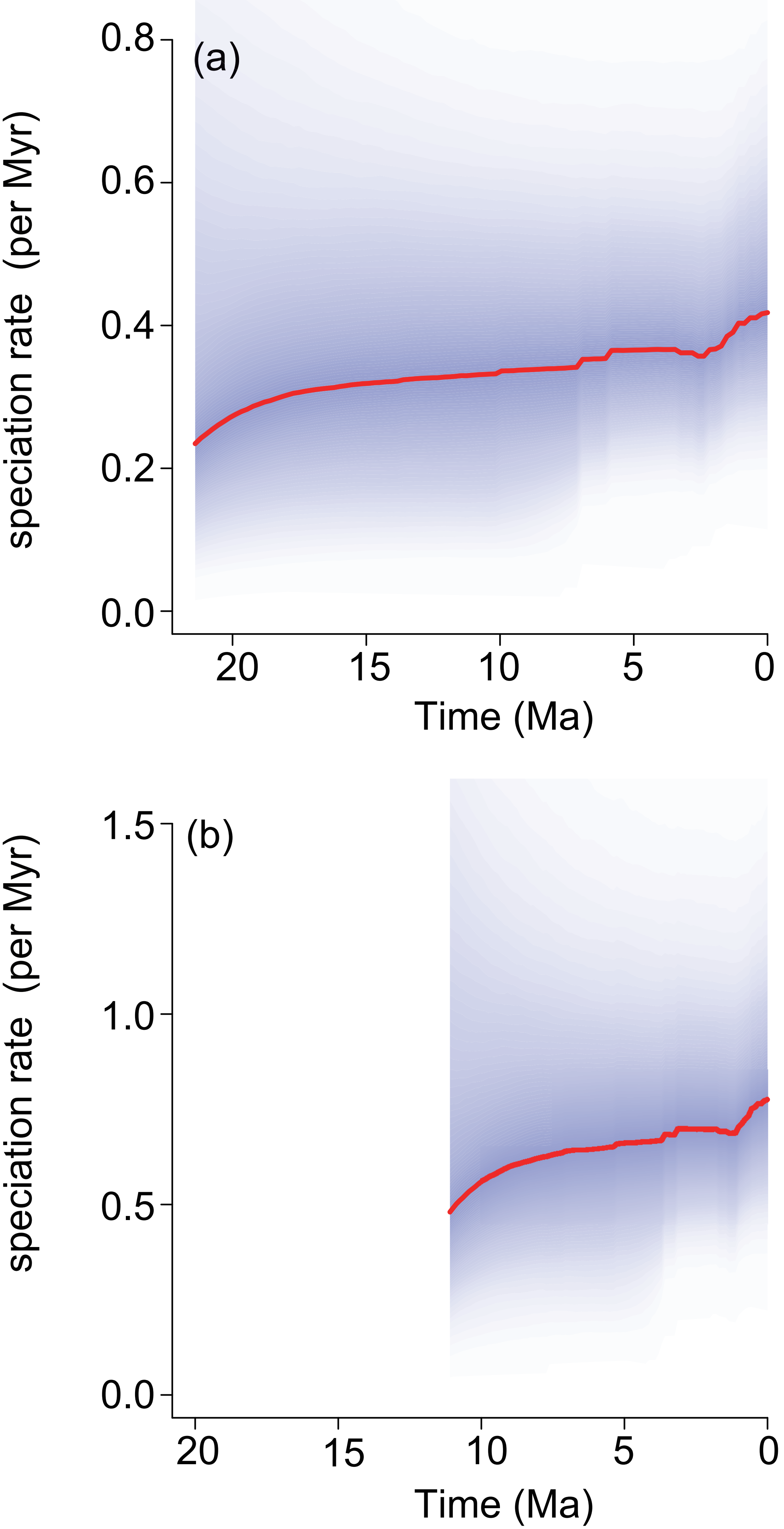

(a)
speciation rate (per Myr)
0.8
0.6
0.4
0.2
0.0
20
15
10
5
0
Time (Ma)
(b)
speciation rate (per Myr)
1.5
1.0
0.5
0.0
20
15
10
5
0
Time (Ma)
